# Supplementary material for: Electric-field control of field-free spin-orbit torque switching via laterally modulated Rashba effect in Pt/Co/AlOx structures
Source: Nat Commun. 2021 Dec 7;12:7111. doi: 10.1038/s41467-021-27459-2 (PMC8651747; doi:10.1038/s41467-021-27459-2)
Supplement: Supplementary file 1 — Supplementary Information [file 41467_2021_27459_MOESM1_ESM.pdf]

Supplementary Information

Electric field control of field-free spin-orbit torque switching via laterally modulated Rashba effect in Pt/Co/AlO_x structures

Min-Gu Kang[1†], Jong-Guk Choi[1†], Jimin Jeong[1], Jae Yeol Park[1], Hyeon-Jong Park[2], Taehwan Kim[3], Taekhyeon Lee[4], Kab-Jin Kim[4], Kyoung-Whan Kim[5], Jung Hyun Oh[6], Duc Duong Viet[7], Jong-Ryul Jeong[7], Jong Min Yuk[1], Jongsun Park[3], Kyung-Jin Lee[4] and Byong-Guk Park[1★]

[1]*Department of Materials Science and Engineering, KAIST, Daejeon 34141, Korea*

[2]*KU-KIST Graduate School of Converging Science and Technology, Korea University, Seoul 02841, Korea*

[3]*School of Electrical Engineering, Korea University, Seoul 02841, Korea*

[4]*Department of Physics, KAIST, Daejeon 34141, Korea*

[5]*Center for Spintronics, Korea Institute of Science and Technology, Seoul 02792, South Korea*

[6]*Department of Materials Science and Engineering, Korea University, Seoul 02841, Korea*

[7]*Department of Materials Science and Engineering, Chungnam National University, Daejeon 34134, Korea*

[†] These two authors equally contributed to this work.

★ Correspondence to: bgpark@kaist.ac.kr (B.-G.P.)

Supplementary Note 1. Electron energy loss spectroscopy analysis of Pt/Co/AlO_x/TiO_2 structure.

To investigate the effect of the gate voltage on oxygen concentration in Pt (5nm)/Co (1.4nm)/AlO_x (2 nm)/TiO_2 (40 nm) samples, we perform scanning transmission electron microscopy (STEM) and electron energy loss spectroscopy (EELS) measurements, which provides a hint for the lateral asymmetry caused by the gate voltage. Before preparing a cross-sectional sample using focused ion beam (FIB) technique, we applied an asymmetric gate voltage ($V_{G,L}$ = 0V, $V_{G,R}$ = +8V), $\Delta V_G < 0$. Figure S1a shows the STEM image and elemental maps of the oxygen obtained from EELS, in which the position A (B) represents the region of the gate oxide located just below the edge of the gate electrode $V_{G,L}$ ($V_{G,R}$). This demonstrates that the oxygen concentration is increased at the position B where a $V_{G,R}$ of +8V was applied as compared to the position A. This indicates that the ΔV_G induces the oxygen ion redistribution depending on its polarity, resulting in the oxygen concentration gradient (Fig. S1b). Moreover, Figure S1c shows the EELS spectra of the Ti L edges measured at the positions A and B, indicating that a positive V_G causes a valence change of the Ti ion into a higher oxidation state, and thus Ti $L_{3,2}$ peaks move toward higher energy [S1]. From the oxygen EELS intensity, the stoichiometries are qualitatively estimated as $TiO_{1.84}$ on the left and $TiO_{2.16}$ on the right.

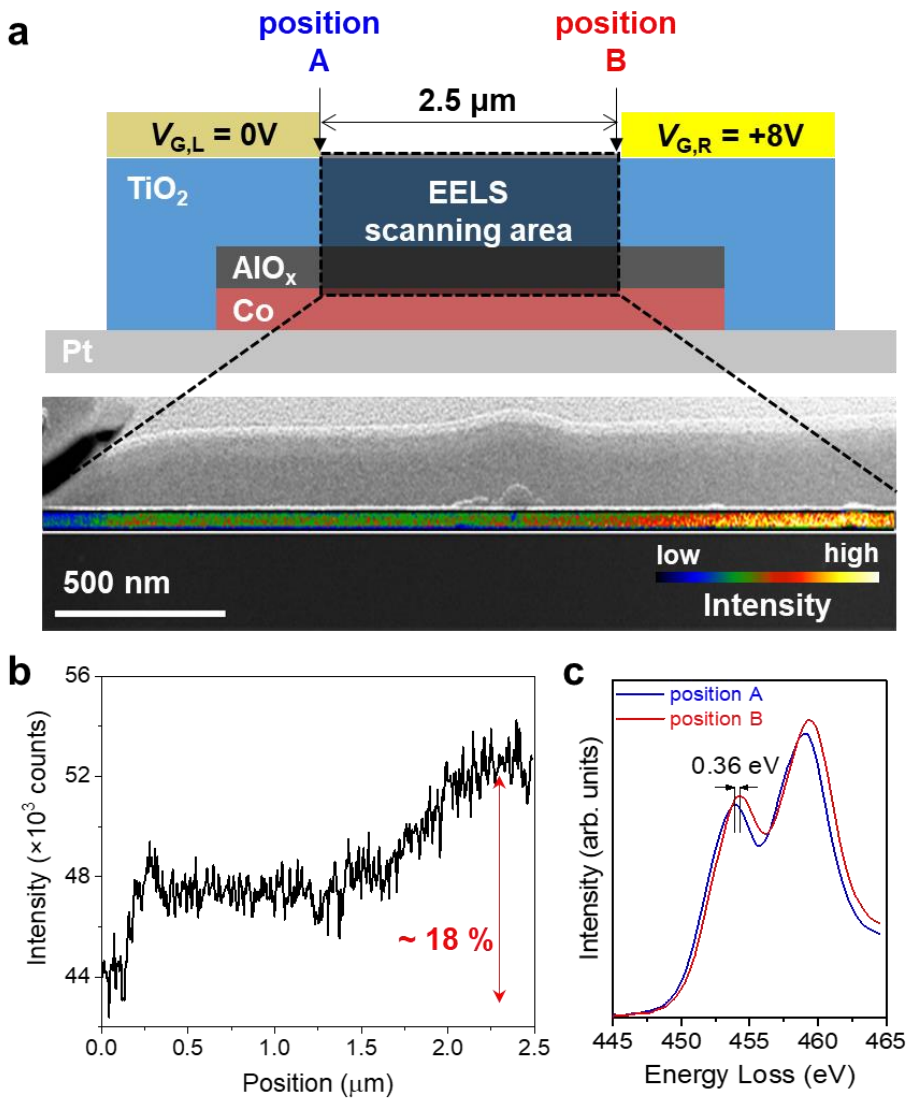

Figure S1. Electron energy loss spectroscopy analysis. a, STEM image and EELS mapping of oxygen in the TiO_2 layer of a Pt(5nm)/Co(1.4nm)/AlO_x(2 nm)/TiO_2(40 nm) structure. **b**, EELS intensity of oxygen in TiO_2 as a function of the position indicated in Fig. S1a. **c**, EELS spectra of the of Ti $L_{3,2}$ edges at the positions A and B.

Supplementary Note 2. Non-volatility of the electric-field effect

To demonstrate the non-volatile behavior of our device, we examined how long the voltage-controlled magnetic anisotropy (VCMA) effect persists in a Pt (5 nm)/Co (1.4 nm)/AlO_x (2 nm)/TiO_2 (40 nm) structure, which is the same sample used in Fig. 1 of the main text. **Figure S2a** shows the VCMA effect of the sample; coercivity (B_c) is reduced when a V_G of +8V (equivalent to 2.5 MV/cm) is applied. To demonstrate the retention of the VCMA effect, we repeatedly measured the B_c over a week, where the measurement was performed every hour on the first day and once a day thereafter. **Figure S2b** shows that the reduced B_c remains almost the same over the measurement time, demonstrating the retention time of the VCMA effect exceeds 7 days, corresponding to 6×10^5 seconds.

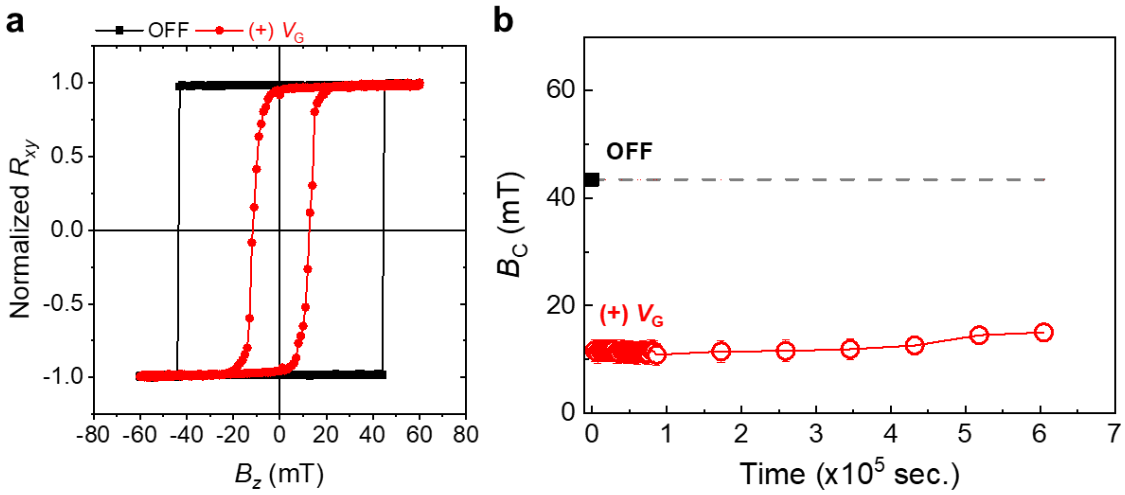

Figure S2. Non-volatility of voltage-controlled magnetic anisotropy in Pt/Co/AlO_x/TiO_2 samples. (a) Normalized anomalous Hall resistance (R_{xy}) versus out-of-plane magnetic field (B_z) before and after applying V_G = +8 V. (b) Coercivity (B_c) versus measurement time.

Supplementary Note 3. Reconfigurable logic operation using a single device

Figures 1 and 3 in the main text show the electrical control of spin-orbit torque switching polarity in a reversible and non-volatile manner. This allows us to demonstrate reconfigurable logic operations using a single device, where a gate voltage ($V_{G,L}$ or $V_{G,R}$) and input current (I_{IN}) are used as two input parameters (Fig. S3a). Figure S3b shows the device performance; the magnetization switching occurs at a current of ±10 mA, and the switching polarity is reversed by changing the sign of ΔV_G. Based on the device, we construct a truth table, as shown in Figs. S3c,d, demonstrating that two logic operations are possible depending on the input parameters; first, the device operates the XOR function with input parameters of I_{IN} and $V_{G,L}$ (Fig. S3c). Here, we define the positive values of the inputs (I_{IN} = +15 mA, $V_{G,L}$ = +10 V) as digital input '1', and their negative values (I_{IN} = −15 mA, $V_{G,L}$ = −10 V) as digital input '0'. They determine the digital output of the device, the magnetization direction; UP [DOWN] magnetization ($R_{xy} > 0$) [($R_{xy} < 0$)] corresponds to digital output '1' ['0']. Second, it operates the AND function with input parameters of I_{IN} and $V_{G,R}$ when the device is initialized as the '0' state (Fig. S3d). For a AND gate, we define the inputs of I_{IN} = +15 mA, $V_{G,L}$ = +10 V as digital input '1', and those of I_{IN} = +5 mA, $V_{G,L}$ = −10 V as digital input '0'. The different logic operations can be achieved by changing one input parameter, demonstrating a programmable logic operation in the SOT device with two side gates.

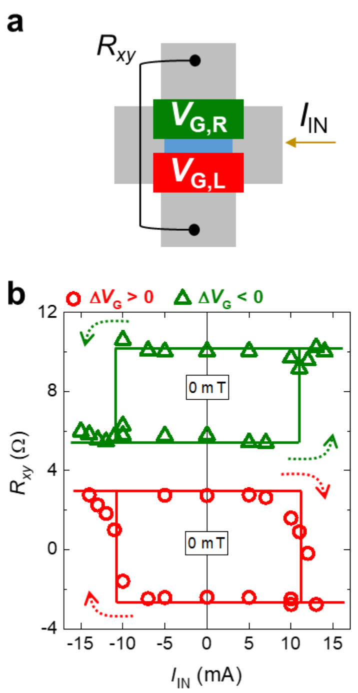

c

Logic input		Logic output
I_{IN} (A)	V_G (B)	R_{xy} ($m_{initial}$ independent)
- 15 mA (0)	-10 $V_{G,L}$ ($\Delta V_G<0$) (0)	0
+ 15 mA (1)	-10 $V_{G,L}$ ($\Delta V_G<0$) (0)	1
- 15 mA (0)	+10 $V_{G,L}$ ($\Delta V_G>0$) (1)	1
+ 15 mA (1)	+10 $V_{G,L}$ ($\Delta V_G>0$) (1)	0

d

Logic input		Logic output
I_{IN} (A)	V_G (B)	R_{xy} ($m_{initial}\downarrow$)
+ 5 mA (0)	-10 $V_{G,R}$ ($\Delta V_G>0$) (0)	0
+ 15 mA (1)	-10 $V_{G,R}$ ($\Delta V_G>0$) (0)	0
+ 5 mA (0)	+10 $V_{G,R}$ ($\Delta V_G<0$) (1)	0
+ 15 mA (1)	+10 $V_{G,R}$ ($\Delta V_G<0$) (1)	1

Figure S3. Reconfigurable logic operations of a single SOT device. a, Schematic of the device, in which gate voltage ($V_{G,L}$ or $V_{G,R}$) and input current I_{IN} are used as two input parameters. **b**, Current-induced switching of the device according to ΔV_G under +10 V of $V_{G,L}$ ($\Delta V_G > 0$, red) or $V_{G,R}$ ($\Delta V_G < 0$, green). **c-d**, Logic truth tables constructed using the results in Fig. S3b. XOR function (c) and AND function (d).

Supplementary Note 4. In-plane harmonic Hall voltage analysis of the spin-orbit torque

Landau-Lifshitz-Gilbert equation including out-of-plane spin-orbit torque (SOT) terms is given as [S2-S4]

$$\frac{\partial \mathbf{m}}{\partial t} = -\gamma \mathbf{m} \times \left(\boldsymbol{B}_{\mathbf{eff}} + B_{DLT}^{y}(\mathbf{m} \times \mathbf{y}) + B_{FLT}^{y}\mathbf{y} + B_{DLT}^{z}(\mathbf{m} \times \mathbf{z}) + B_{FLT}^{z}\mathbf{z}\right) + \alpha \mathbf{m} \times \frac{\partial \mathbf{m}}{\partial t} \quad \text{(S1)},$$

where $\boldsymbol{B}_{\mathbf{eff}}$ includes the anisotropy (B_{ani}), demagnetization (B_{dem}), exchange, and external fields; α is the damping constant; γ is the gyromagnetic ratio; B_{DLT}^{y} (B_{FLT}^{y}) and B_{DLT}^{z} (B_{FLT}^{z}) are the damping-like (field-like) SOT effective field from y-SOT and z-SOT, respectively. Here, we define the unit vector **x** as the current direction and **z** as the direction perpendicular to the film plane. We assume that inversion symmetry is broken in both the z- and y-directions, generating y-SOT and z-SOT, respectively. Figure S4a shows the effective fields due to y-SOT and z-SOT acting on the magnetization.

The current-induced effective field ($\Delta\boldsymbol{B}$) due to the inversion asymmetry along the z- and y-directions is given by [S5]

$$\Delta\boldsymbol{B} = \left(\Delta B_x, \Delta B_y, \Delta B_z\right) = (-B_{\mathrm{DLT}}^{y}\cos\theta_0 + B_{\mathrm{DLT}}^{z}\sin\theta_0\sin\varphi_0\,,\; B_{\mathrm{FLT}}^{y} + B_{Oe} -$$

$$B_{\mathrm{DLT}}^{z}\sin\theta_0\cos\varphi_0\,, B_{\mathrm{DLT}}^{y}\sin\theta_0\cos\varphi_0 + B_{\mathrm{FLT}}^{z}) \quad \text{(S2)},$$

where B_{Oe} is the Oersted field, θ and φ are the polar and azimuthal angles of the magnetization direction, respectively. The subscript "0" denotes the equilibrium state of magnetization.

When applying a sinusoidal oscillating current ($I = \Delta I \sin\omega t$) with frequency ω, the Hall voltage V_{xy} is expressed as,

$$V_{xy} = R_{xy}I = \left(R_{xy,0} + \frac{dR_{xy}}{dI}\Delta I\sin\omega t\right)\Delta I\sin\omega t \quad \text{(S3)},$$

where R_{xy} is the Hall resistance. In normal-metal/ferromagnet/oxide structure, the R_{xy} is

$$R_{xy} = R_{AHE}\cos\theta + R_{PHE}\sin^2\theta\sin 2\varphi \quad \text{(S4)},$$

where R_{AHE} (R_{PHE}) is the anomalous Hall resistance (planar Hall resistance). The spin torque

from $\Delta I \sin\omega t$ induces the perturbation of magnetization in polar angle ($\Delta\theta \sin\omega t$) and azimuthal angle ($\Delta\varphi \sin\omega t$). Therefore, the R_{xy} can be expanded as,

$$R_{xy} = R_{xy,0} + \frac{dR_{xy}}{dI}\Delta I \sin\omega t \approx R_{AHE}(\cos\theta_0 - \Delta\theta \sin\omega t \sin\theta_0) +$$

$$R_{PHE}(\sin^2\theta_0 \sin 2\varphi_0 + \Delta\theta \sin\omega t \sin 2\theta_0 \sin 2\varphi_0 + 2\Delta\varphi \sin\omega t \sin^2\theta_0 \cos 2\varphi_0) \quad \text{(S5)}.$$

When in-plane component of an external magnetic field (B_{ext}) with polar angle θ_B and azimuthal angle φ_B is sufficiently larger than the demagnetization field B_{dem} ($|B_{dem}| \ll |B_{ext}| \sin\theta_B$ and $\varphi_0 \approx \varphi_B$), the perturbation angles become [S5]

$$\Delta\theta = \frac{\cos\theta_0(\Delta B_x \cos\varphi_B + \Delta B_y \sin\varphi_B) - \sin\theta_0 \Delta B_z}{(B_{ani} - B_{dem}\sin^2\varphi_B)cos2\theta_0 + B_{ext}cos(\theta_B - \theta_0)} \quad \text{(S6)}$$

$$\Delta\varphi = \frac{-\Delta B_x \sin\varphi_B + \Delta B_y \cos\varphi_B}{-B_{dem}\sin\theta_0 \cos 2\varphi_B + B_{ext}\sin\theta_B} \quad \text{(S7)}.$$

By substituting the Eq.(S2) and Eqs. (S5-S7) into Eq.(S3), the Hall voltage and each harmonic Hall resistance ($R_{xy}^{0\omega}$, $R_{xy}^{1\omega}$, and $R_{xy}^{2\omega}$) can be rewritten as

$$V_{xy} = (\tfrac{1}{2}R_{xy}^{0\omega} + R_{xy}^{1\omega}\sin\omega t - \tfrac{1}{2}R_{xy}^{2\omega}\cos 2\omega t)\,\Delta I \quad \text{(S8)}.$$

$$R_{xy}^{0\omega} = A + B \quad \text{(S9)}.$$

$$R_{xy}^{1\omega} = R_{xy,0} \quad \text{(S10)}.$$

$$R_{xy}^{2\omega} = A + B \quad \text{(S11)}.$$

$$A = (-R_{AHE}\sin\theta_0 + 2R_{PHE}\sin 2\theta_0 \sin 2\varphi_H)$$

$$\times \frac{\left(-B_{FLT}^{z}\sin\theta_0 - B_{DLT}^{y}\cos\varphi_H + (B_{FLT}^{y} + B_{Oe})\cos\theta_0 \sin\varphi_B\right)}{(B_{ani} - B_{dem}\sin^2\varphi_B)cos2\theta_0 + B_{ext}cos(\theta_B - \theta_0)}$$

$$B = 2R_{PHE}\sin^2\theta_0 \cos 2\varphi_H \frac{B_{DLT}^{y}\cos\theta_0 \sin\varphi_B + (B_{FLT}^{y} + B_{Oe})\cos\varphi_B - B_{DLT}^{z}\sin\theta_0}{-B_{dem}\sin\theta_0 \cos 2\varphi_B + B_{ext}\sin\theta_B}$$

In particular, when applying an in-plane magnetic field ($\theta_B = \frac{\pi}{2}$) sufficient to make the magnetization direction aligned in the plane ($\theta_0 \approx \frac{\pi}{2}$), B_{FLT}^{y} and B_{DLT}^{z} (B_{DLT}^{y} and B_{FLT}^{z}) only changes the azimuthal (polar) angle of magnetization, as shown in Fig. S4b. In such condition,

the second harmonic Hall resistance $R_{xy}^{2\omega}$ becomes

$$R_{xy}^{2\omega}(\varphi_B) = \left(R_{\text{AHE}}\frac{B_{\text{DLT}}^{y}}{B_{\text{eff}}} + R_{\nabla T}^{2\omega}\right)\cos\varphi_B + 2R_{\text{PHE}}\frac{B_{\text{FLT}}^{y}+B_{\text{Oe}}}{B_{\text{ext}}}(2\cos^3\varphi_B - \cos\varphi_B) - 2R_{\text{PHE}}\frac{B_{\text{DLT}}^{z}}{B_{\text{ext}}}\cos 2\varphi_B + R_{\text{AHE}}\frac{B_{\text{FLT}}^{z}}{B_{\text{eff}}} \quad \text{(S12)},$$

Where thermal contribution ($R_{\nabla T}^{2\omega}$) to $R_{xy}^{2\omega}$ is included. Equation (S12) is the same as Eq. (1) in the main text.

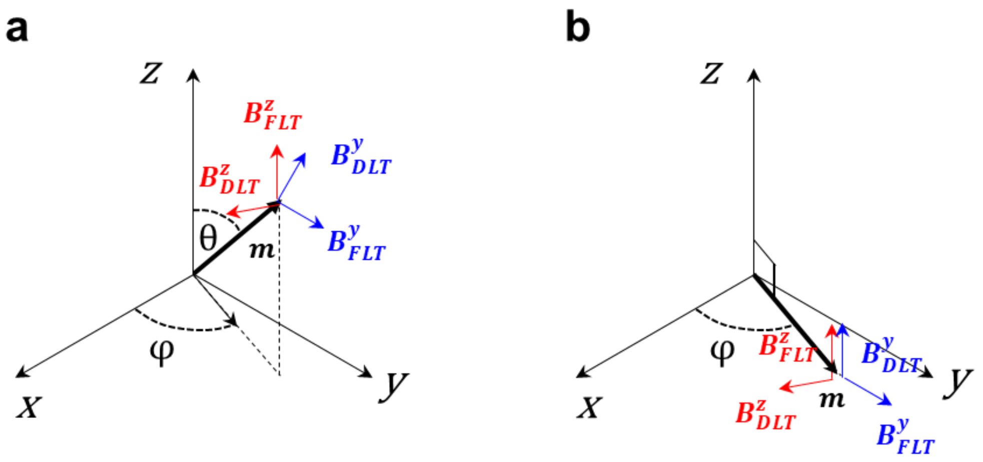

Figure S4. Effective fields due to y-SOT and *z*-SOT. a-b, The effective field directions acting on the arbitrary magnetization direction (**a**) and $\theta = \frac{\pi}{2}$ (**b**).

Supplementary Note 5. Hysteresis loops shift of anomalous Hall resistance

We measure the anomalous Hall resistance R_{xy} with a d.c. current $J_{d.c}$ as a function of the out-of-plane magnetic field B_z. The R_{xy} vs. B_z hysteresis loop will shift when z-SOT is present [S3,S6]. Figures S5a shows the hysteresis loop in Pt/Co/AlO_x/TiO_2 structures (TiO_2 sample) depending on the ΔV_G. When $\Delta V_G > 0$ ($V_{G,L}$ = +8V, $V_{G,R}$ = −8V), the hysteresis loop shifts to the positive B_z direction for a positive $J_{d.c}$, while it is opposite for $\Delta V_G < 0$. Figure S5b shows that the ΔB_S gradually increases with $J_{d.c}$ without a threshold current. Here, ΔB_S is the amount of the hysteresis loop shift, defined $\Delta B_S(I_{d.c.}) = [|B_C^+(I_{d.c.})| - |B_C^-(I_{d.c.})|]/2$, where $B_C^\pm$ are positive and negative coercive fields. Figures S5c,d show the results of the ZrO_2 sample, demonstrating the hysteresis shift direction of the ZrO_2 sample is opposite to that of the TiO_2 sample. This is consistent with the SOT switching and harmonic measurement results in the main text.

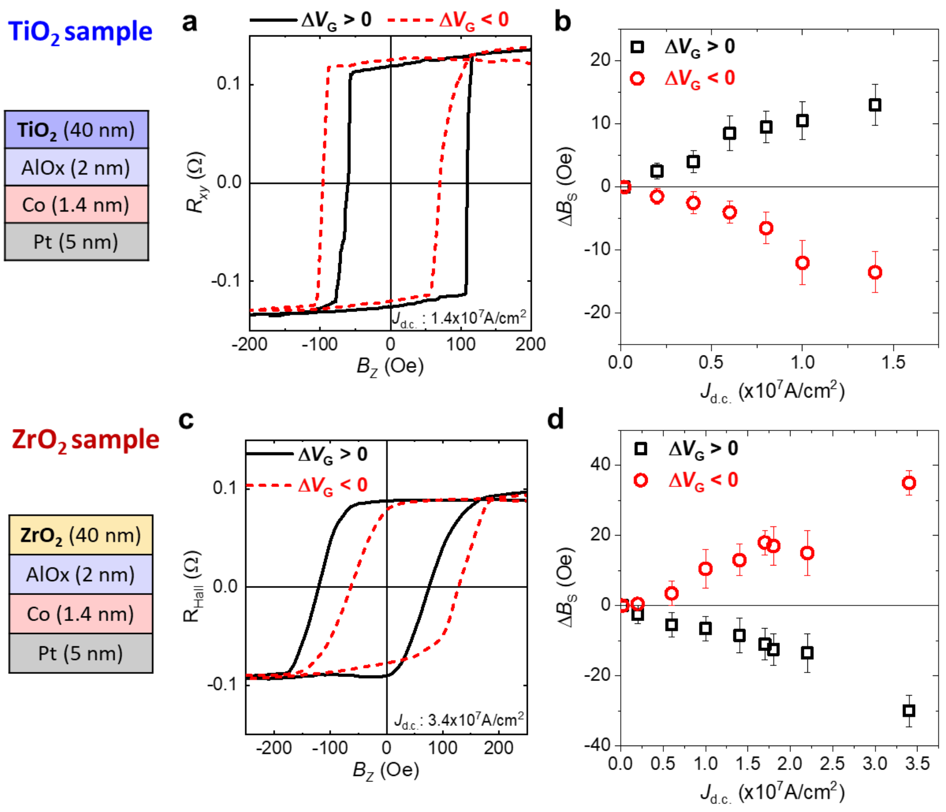

Figure S5. Hysteresis loop shift of R_{xy} in Pt/Co/AlO$_x$/gate oxide structure a, R_{xy} versus B_z curves in the TiO_2 sample for $\Delta V_G > 0$ ($V_{G,L} = +8$ V, $V_{G,R} = -8$ V) (black line) and $\Delta V_G < 0$ ($V_{G,L} = -8$ V, $V_{G,R} = +8$ V) (red dash line). **b**, ΔB_S versus $I_{d.c.}$ curves in the TiO_2 sample for $\Delta V_G > 0$ (black square) and $\Delta V_G < 0$ (red circle). **c**, R_{xy} versus B_z curves in the ZrO_2 sample for $\Delta V_G > 0$ ($V_{G,L} = +10$ V, $V_{G,R} = -10$ V) (black line) and $\Delta V_G < 0$ ($V_{G,L} = -10$ V, $V_{G,R} = +10$ V) (red dash line). **d**, ΔB_S versus $I_{d.c.}$ curves in the ZrO_2 sample for $\Delta V_G > 0$ (black square) and $\Delta V_G < 0$ (red circle). The error bars in figures are the standard deviation of the mean values measured five times.

Supplementary Note 6. Gate voltage dependence of field-free switching current density

To investigate the correlation between the field-free switching current density (J_{SW}) and ΔV_G, we measure J_{SW} in a Pt (5 nm)/Co (1.4 nm)/AlO_x (2 nm)/ZrO_2 (40 nm) structure with different ΔV_G's of +10, +12, +14 V, equivalent to 2.5, 3.0, and 3.5 MV/cm, respectively (Fig. S6a), which are compared to that measured for $\Delta V_G = 0$ and $B_x = 20$ mT. Figure S6b shows that J_{SW} gradually reduces as ΔV_G increases, corroborating that *z*-SOT increases with larger ΔV_G. This is also consistent with theoretical result [S4]. Furthermore, we confirm that the J_{SW} of the sample with $\Delta V_G = 0$ gradually decreases as B_x increases (Fig. S6c).

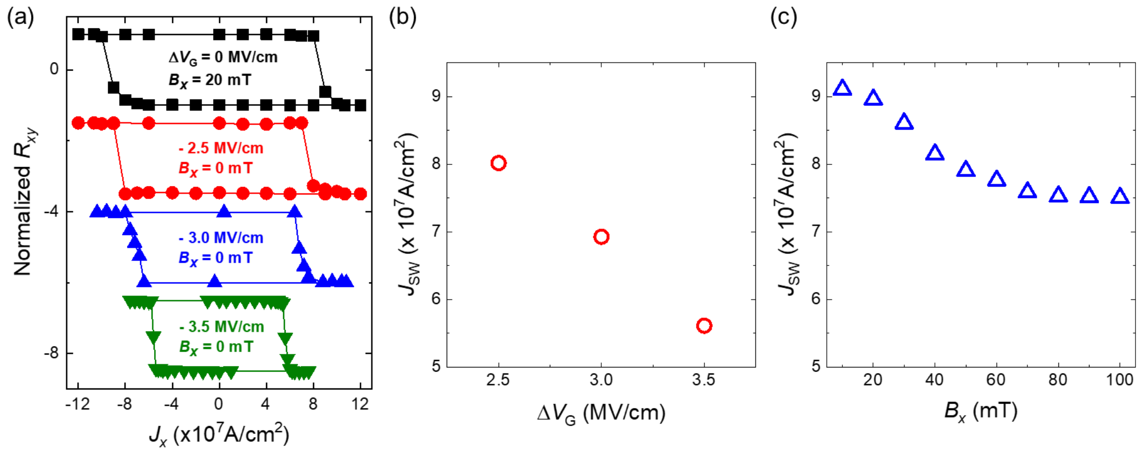

Figure S6. Gate voltage dependence of field-free switching current density. a, Field-free SOT switching in Pt/Co/AlO_x/ZrO_2 samples with different ΔV_G's of 0, 2.5, 3.0, and 3.5 MV/cm. **b,** Switching current density (J_{SW}) vs. ΔV_G. **c,** the J_{SW} of the sample with $\Delta V_G = 0$ as a function of in-plane magnetic field (B_x).

Supplementary Note 7. Microscopic observation of the field-free switching

We performe magneto-optical Kerr effect (MOKE) measurements to examine the magnetization reversal characteristics during field-free SOT switching in Pt (5 nm)/Co (1.4 nm)/AlO_x (2 nm)/ZrO_2 (40 nm) samples. Here, we use a device having a single gate on the right side, where a $V_{G,R}$ of +3.5 MV/cm (+14 V) is applied, thus $\Delta V_G < 0$. The domain images are captured after a current pulse (J_x) of $+5.4 \times 10^7 A/cm^2$ without an external magnetic field. Figure S7a shows an initial state, where the central bright area indicates magnetization pointing in the +z direction. Figures S7b-f show the up-to-down switching sequence; a down domain corresponding to the dark contrast first nucleates at the center of the ferromagnetic island and it then propagates outward by repeatedly injecting current pulses. We note that the ΔV_G-induced z-SOT is maximum at the center region (or the edge of the gate electrode) where the reversed domain nucleates. Figures S7g-l show the down-to-up switching, which is the same behavior as the up-to-down switching. This result rules out the contribution of the Oersted field effect [S7], corroborating that the field-free SOT switching is dominantly governed by the ΔV_G-induced z-SOT in our devices.

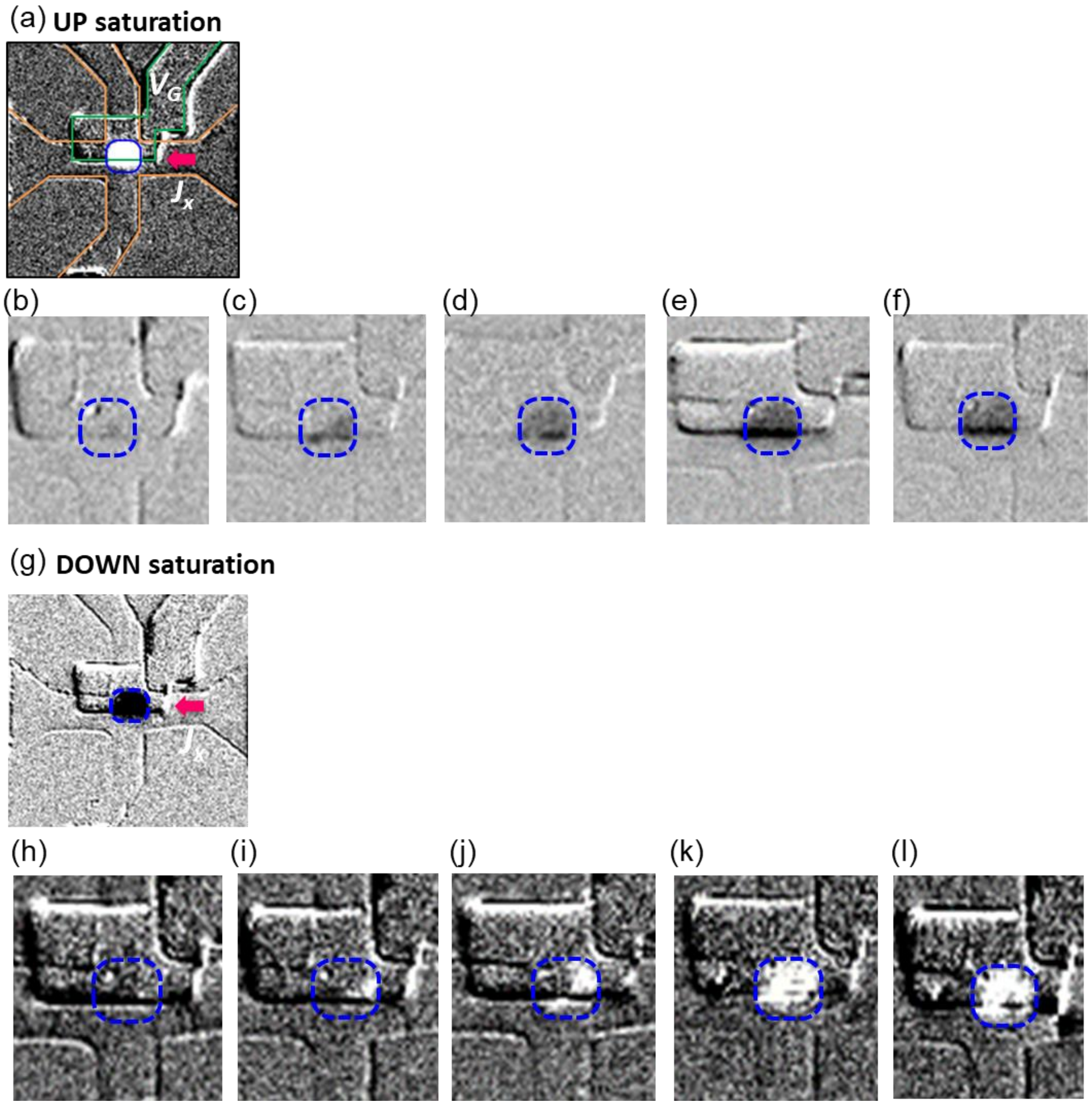

Figure S7. MOKE images of field-free switching in the Pt/Co/AlO_x/ZrO_2 sample. a, Initial UP magnetization state defined by applying a perpendicular magnetic field B_z of 60 mT. **b-f**, Sequential MOKE images after successive current pulses of + 5.4×10^7A/cm^2. **g**, Initial DOWN magnetization state defined by applying a B_z of – 60 mT. **h-l**, Sequential MOKE images after successive current pulses of – 5.4×10^7A/cm^2.

Supplementary Note 8. Spin-orbit torque measurements in Pt/Co/AlOx/ZrO2 sample

We perform the in-plane harmonic Hall measurements [S8] for a Pt/Co/AlO_x/ZrO_2 structure (ZrO_2 sample). Figures S8a-c show the $\cos\varphi$, $2\cos^3\varphi - \cos\varphi$, and $\cos2\varphi$ components of the $R_{xy}^{2\omega}$ as a function of the B_{eff} or B_{ext}, which correspond to B_{DLT}^{y}, B_{FLT}^{y} and B_{DLT}^{z}, respectively. For the ZrO_2 sample, the $\cos2\varphi$ component or B_{DLT}^{z} only appears when an asymmetric V_G is applied, similar to the TiO_2 sample (Fig. 3 of the main text). However, the sign of B_{DLT}^{z} in the ZrO_2 sample is opposite to that of the TiO_2 sample, which is consistent with field-free switching results shown in Fig. 3 of the main text. The estimated SOT-induced effective SOT fields according to the gate voltage conditions are summarized in Table S1.

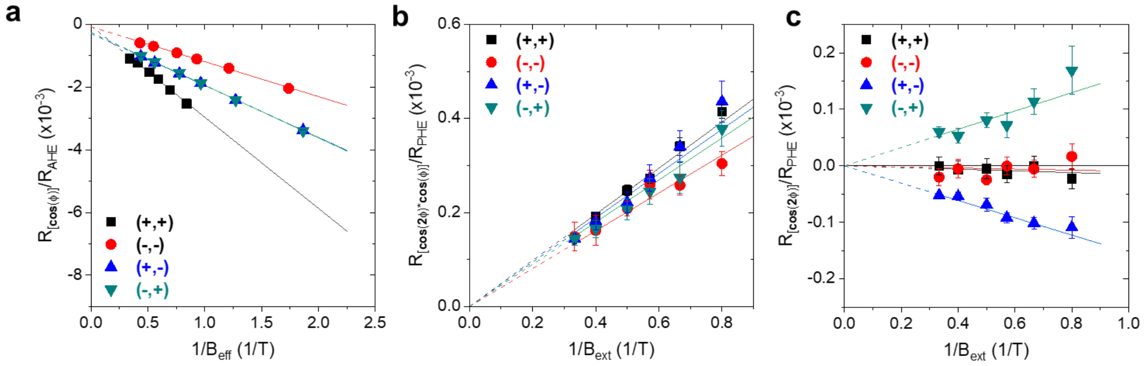

Figure S8. Harmonic spin-orbit torque measurements in Pt/Co/AlOx/ZrO2. a, $\cos\varphi$ component of $R_{xy}^{2\omega}$ versus $1/B_{\mathrm{eff}}$. **b**, $(2\cos^3\varphi - \cos\varphi)$ component of $R_{xy}^{2\omega}$ versus $1/B_{\mathrm{ext}}$. **c**, $\cos2\varphi$ component of $R_{xy}^{2\omega}$ versus $1/B_{\mathrm{ext}}$. The error bars are due to the uncertainty of the fitting of the $R_{xy}^{2\omega}$ versus φ curves to Eq. (1).

Table S1. SOT effective fields depending on gate voltage conditions in ZrO_2 samples.

Effective field	(+,+)	(−,−)	(+,−)	(−,+)
$B_{\mathrm{FLT}}^{\mathrm{y}}$ (mT)	0.49 ± 0.02	0.41 ± 0.02	0.46 ± 0.02	0.46 ± 0.02
$B_{\mathrm{DLT}}^{\mathrm{y}}$ (mT)	− 2.91 ± 0.01	− 1.11 ± 0.01	− 1.67 ± 0.02	− 1.67 ± 0.02
$B_{\mathrm{DLT}}^{\mathrm{z}}$ (mT)	− 0.02 ± 0.001	− 0.01 ± 0.001	− 0.15 ± 0.01	0.15 ± 0.01

Supplementary Note 9. Voltage-controlled magnetic anisotropy (VCMA) effect

We investigate voltage-controlled magnetic anisotropy (VCMA) effect of the Pt (5 nm)/Co (1.4 nm)/AlO_x (2 nm) structures with different gate oxides of TiO_2 (40 nm) or ZrO_2 (40 nm), in which FM Co is fully covered by a single gate. We measure the anomalous Hall resistance (R_{xy}) as a function of an in-plane magnetic field (B_x), allowing us to evaluate the perpendicular magnetic anisotropy field (H_K) and its variation with gate voltage (V_G) [S9-S11]. Figures S9a,b show that the H_K decreases when applying a positive gate voltage (V_G) for both samples regardless of the gate oxide. We further investigate the VCMA effect of the same samples that are used for the SOT switching measurements in the main text with four different gate conditions of $V_G^{(+,+)}$, $V_G^{(-,-)}$, $V_G^{(+,-)}$, and $V_G^{(-,+)}$. Figures S9c-7f show the changes in the coercivity H_C and H_K with V_G, demonstrating that the H_C and H_K increase (decrease) when applying negative (positive) voltages to both left and right gate electrodes simultaneously: $V_G^{(-,-)}$ ($V_G^{(+,+)}$). On the other hand, for the asymmetric voltages $V_G^{(+,-)}$ and $V_G^{(-,+)}$, the H_C and H_K exhibit intermediate values. Moreover, the VCMA effect exhibits the same sign for both samples regardless of the gate oxides.

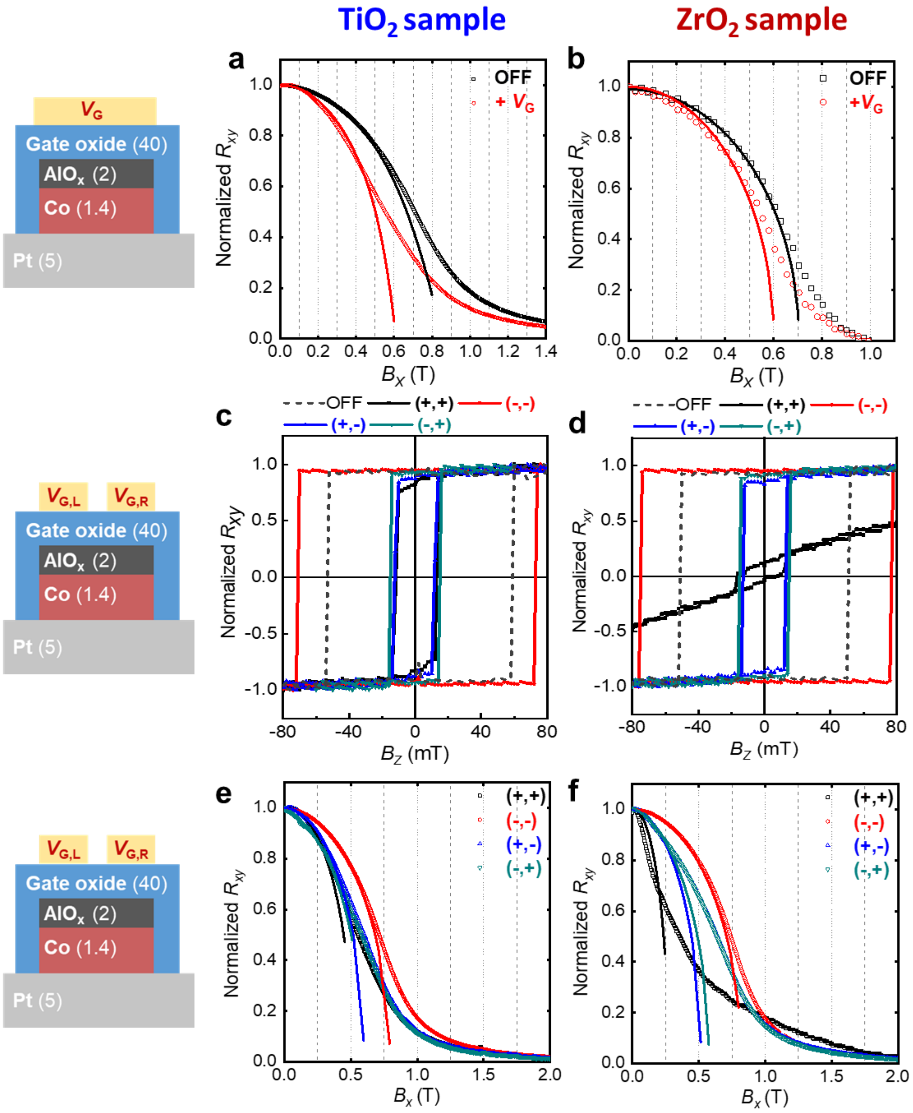

Figure S9. Voltage-controlled magnetic anisotropy measurements. a-b, Normalized anomalous Hall resistance (R_{xy}) as a function of in-plane magnetic field (B_x) under V_G = +8 V for the TiO_2 sample (a) and V_G = +10 V for ZrO_2 samples (b) with a single gate. **c-d,** Normalized R_{xy} as a function of an out-of-plane magnetic field (B_z) under different gate voltages of $V_G^{(+,+)}$, $V_G^{(-,-)}$, $V_G^{(+,-)}$, and $V_G^{(-,+)}$ for the TiO_2 sample (c) and ZrO_2 sample (d). **e-f,** Normalized R_{xy} as a function of B_x under different gate voltages of $V_G^{(+,+)}$, $V_G^{(-,-)}$, $V_G^{(+,-)}$, and $V_G^{(-,+)}$ for the TiO_2 sample (e) and ZrO_2 sample (f).

Supplementary Note 10. COMSOL simulation

It is found that the resistance of the device (R_{xx}) is slightly increased by ~1 % when applying a negative V_G of 10 V. By considering the resistance variation, we calculate the current distribution using COMSOL software. Figure S10a shows the schematic of the device for Pt (5 nm)/Co (1.4 nm), where the dimension of the Pt bottom electrode is 10 μm × 25 μm and the dimension of the Co island is 8 μm × 8 μm, identical to the actual device in our experiments. Here, we assume that the resistance increases only in the area under the gate electrode of 4 μm × 25 μm. Figure S10b shows the distribution of the current density along the *x*-axis and *y*-axis, corresponding to J_x and J_y, when applying a current density of 1.0x10^7 A/cm^2 along the *x*-axis. This shows that there is a shunting J_x flowing into the Co island. Figure S10c shows the lateral current J_x and J_y flowing through the Pt and Co layers, which are averaged in the device area. This demonstrates that J_y is almost 10^6 times smaller than J_x, suggesting that the lateral current distribution is negligibly small in our device.

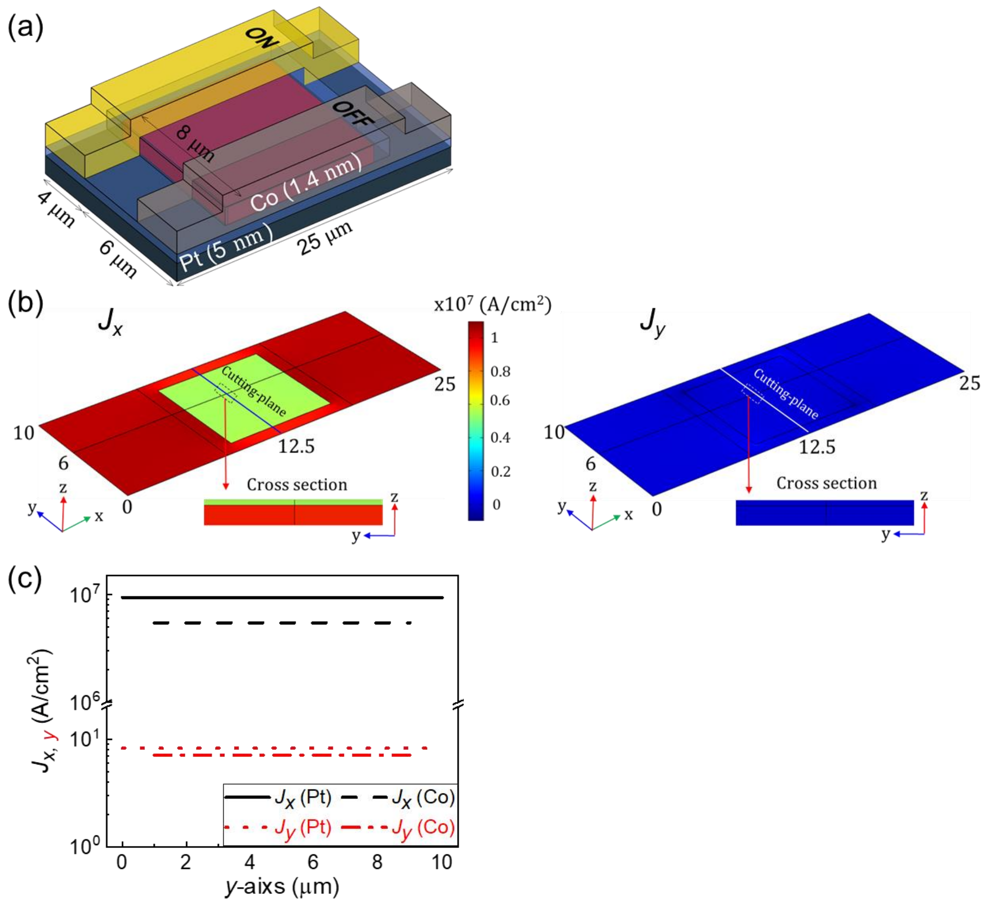

Figure S10. Lateral current distribution in a Pt/Co electrode. a, Schematic of the device structure for the COMSOL simulation. The area where a negative V_G is applied has a larger resistance than the rest of the area. **b**, Distribution of the J_x and J_y. **c**, Averaged values of the J_x and J_y, flowing through Pt and Co layers.

Supplementary Note 11. Variation of damping-like spin-orbit torque with gate voltage

Figure S11 shows the $\cos\varphi$ component of $R_{xy}^{2\omega}$ as a function of $1/B_{\mathrm{eff}}$ depending on V_{G} in Pt (0.5 nm)/Co (2 nm)/AlO_x (2 nm)/gate oxide (40 nm) samples, which corresponds to the change in B_{DLT}^{y} with V_{G}. Unlike the B_{FLT}^{y} (Figs. 4c-d in the main text), the B_{DLT}^{y} and its gate voltage dependence is negligible for the samples regardless of the gate oxide material.

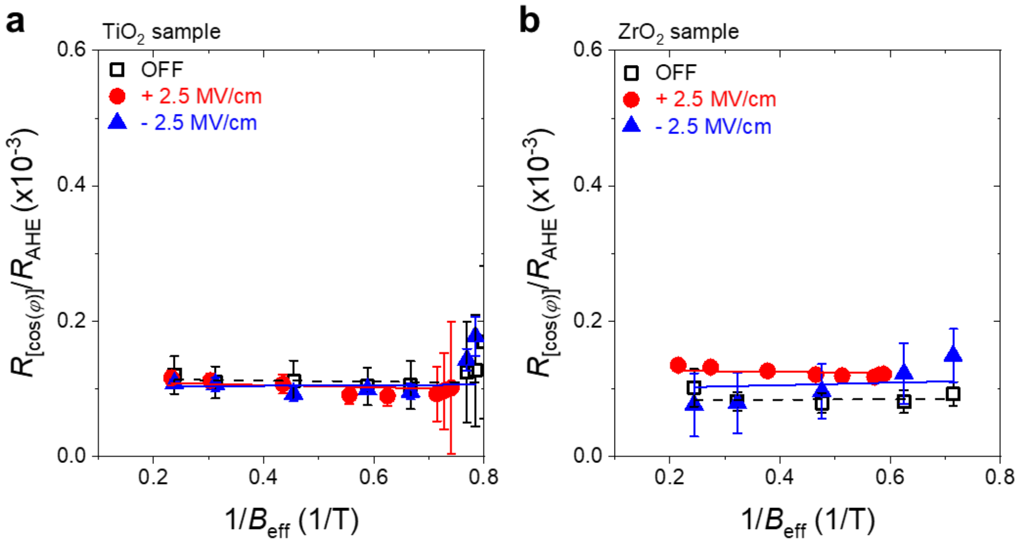

Figure S11. Gate voltage dependence of damping-like SOT. a-b, Damping-like SOT component of $R_{xy}^{2\omega}$ depending on pre-biased gate voltage V_{G} of ±2.5 MV/cm for Pt(0.5 nm)/Co (2 nm)/AlO_x (2 nm)/TiO_2 (40 nm) **(a)** and Pt (0.5 nm)/Co (2 nm)/AlO_x (2 nm)/ZrO_2 (40 nm) **(b)** samples. The error bars are due to the uncertainty of the fitting of the $R_{xy}^{2\omega}$ versus φ curves to Eq. (1).

Supplementary Note 12. Computational details of first-principles calculation and linear response theory

We carry out calculations for the two types of Pt/Co/O structure with and without ∇V_{O} (Figs. 5a-b in the main text). For the case with ∇V_{O}, the lateral oxygen gradient is employed along the *y*-axis (see the right panel of Fig. 5b in the main figure), which mimics our experiment where the lateral oxygen migration is induced by gate voltage. To calculate the ground states, we perform the DFT calculation using the OpenMX package [S12], which is based on the norm-conserving pseudopotential [S13] and the pseudo-atomic localized basis function [S12]. We choose the generalized gradient approximation [S14] for the exchange-correlation functional and use the optimized pseudoatomic orbitals *s2p2d2* for Pt and Co atoms, and *s2p2d1* for O atom. The Pt/Co/O heterostructure consists of $(2 \times 2 \times 1)$ slab supercell where the in-plane lattice constant of cubic unit cell is the experimental value of 3.1349Å for the BCC Pt. In *y*- and *z*-directions of the supercell, 15Å-thick vacuums are employed. We use $(12 \times 1 \times 1)$ *k*-point mesh.

Using the ground state solution, we calculate a non-equilibrium spin density using the linear response theory. The perturbed Hamiltonian with an external electric field is given by

$$\mathcal{H}_{ext}(t) = \frac{\mathbf{p}}{2m} \cdot |e|\mathbf{A}(t) + |e|\mathbf{A}(t) \cdot \frac{\mathbf{p}}{2m} = -\boldsymbol{\mathcal{J}} \cdot \mathbf{A}, \qquad \text{(S13)}$$

where $\mathbf{p}$ is the momentum operator, $\boldsymbol{\mathcal{J}}$ is the current operator, and $\mathbf{A}(t) = -\hat{\mathbf{x}}\mathcal{E} \sin \omega_p t/\omega_p$ is the vector potential with an electric field $\mathcal{E}$ in the x-axis. Considering that our calculation is for DC case, we set a frequency ω_p to be zero at the final stage of calculation. To calculate a non-equilibrium spin density, we introduce the lesser Green's function [S15, S16] as

$$G^{<}(\mathbf{k};t) = -\frac{1}{i\hbar}\left[\rho^{(0)}(\mathbf{k}) + \rho^{(1)}(\mathbf{k},t) + \cdots\right], \qquad (S14a)$$

$$\rho^{(0)}(\mathbf{k}) = \frac{i}{2\pi}\int dE f_0(E) g^C(\mathbf{k},E), \qquad (S14b)$$

$$\rho^{(1)}(\mathbf{k},t) = \frac{i}{2\pi}\int dE f_0(E)\int d\omega e^{-i\omega t}$$

$$\times [g^R(\mathbf{k},E+\hbar\omega)U(\omega)g^C(\mathbf{k},E) + g^C(\mathbf{k},E)U(\omega)g^A(\mathbf{k},E-\hbar\omega)], \quad \text{(S14}c\text{)}$$

where f_0 is the Fermi-Dirac distribution function, and g^R (g^A) is an unperturbed retarded (advanced) Green's function with $g^C = g^R - g^A$, $U(\omega) = 1\,/\,(2\pi i)\int dt\mathcal{H}_{ext}(t)e^{i\omega t}$ is a Fourier component of the perturbed Hamiltonian, an unperturbed retarded Green's function is $g^R(\mathbf{k},E) = [E - \mathcal{H}_0 + i\Gamma]^{-1}$, $\mathcal{H}_0$ is the ground state Hamiltonian with the atomic orbital basis, and Γ is a constant level broadening with the value of $0.25\ m$eV [S17]. Finally, a non-equilibrium spin density is given by

$$\delta s(t) = \sum_{\mathbf{k}} \mathrm{Tr}_{\mathrm{spin}}\{\boldsymbol{\sigma}\rho^{(1)}(\mathbf{k},t)\}, \quad \text{(S15)}$$

where $\boldsymbol{\sigma}$ is the Pauli spin matrix. We use the $\mathbf{k}$-point mesh of $(82\times 1\times 1)$ for the summation over the Brillouin zone in Eq. (S15) and confirm that the non-equilibrium spin density is converged with this $\mathbf{k}$-point mesh.

We show that the lateral oxygen gradient generates the Rashba effect, which can be seen by the Rashba splitting in the band structure. The Rashba model Hamiltonian $\mathcal{H}_R$ is given by [S18]

$$\mathcal{H}_R = \alpha_R(\boldsymbol{\sigma}\times\mathbf{k})\cdot\mathbf{v}, \quad \text{(S16)}$$

where α_R is the Rashba constant and $\mathbf{v}$ is the direction along the inversion symmetry breaking. In our experiment, $\mathbf{v}$ has the *y*-component because of the oxygen gradient along the *y*-axis. Assuming $\mathbf{v} = \mathbf{y}$, Eq. (S16) becomes

$$\mathcal{H}_R = \alpha_R(-\sigma_x k_z + \sigma_z k_x). \quad \text{(S17)}$$

Equation (S17) means that there is the energy splitting of σ_z state along the k_x-direction, i.e., the Rashba splitting [S19]. To explicitly demonstrate the Rashba splitting induced by the oxygen gradient, we compute band structures of two types of Pt/Co/O structure with and

without ∇V_o. We plot the band structures along k_x with the magnetization along the $\pm z$ direction [S20, S21]. This magnetization direction sets the σ_z state along the $\mp z$ direction via a strong exchange coupling. Figure S12 shows that the Rashba splitting is present only when there is the lateral oxygen gradient. Combined with the linear response calculation (Figs. 5c-e in the main text), these results show that the lateral oxygen gradient results in the lateral modulation of Rashba effect, which in turn generates the *z*-SOT.

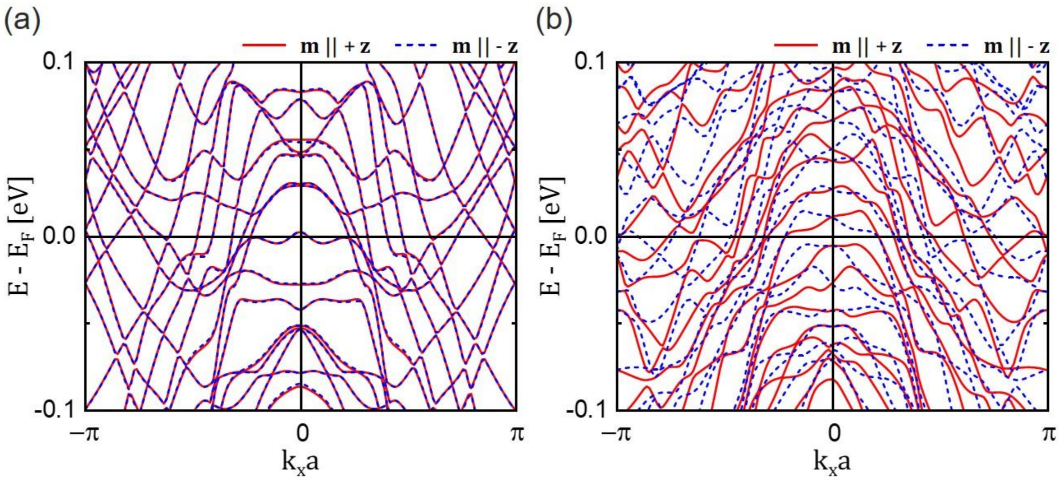

Figure S12. Band structures along the k_x direction of the Pt/Co/O structure. a, without the lateral oxygen gradient. **b**, with the lateral oxygen gradient. The magnetization m is either in the $+z$ direction (red solid line) or in the $-z$ direction (blue dashed line).

Supplementary Note 13. Oxygen gradient dependence of z-component non-equilibrium spin density

We show below ab-initio results that the *z*-component non-equilibrium spin density δs_z is linearly proportional to the lateral oxygen gradient. This means that there is no threshold oxygen gradient to make δs_z nonzero. In other words, δs_z is always nonzero once (even small) oxygen gradient is present. Note that, regardless of its magnitude, nonzero δs_z breaks the switching symmetry and results in the field-free switching.

We construct four different 4×5 Pt/Co/O supercells with different lateral oxygen gradients (Fig. S13a). Each supercell consists of two regions: The region 1 is fully covered by oxygen atoms. On the other hand, the region 2 is partially covered by oxygen atoms; i.e., the number of oxygen vacancies varies from 1 to 4, corresponding to ∇V_O^1, ∇V_O^2, ∇V_O^3, and ∇V_O^4. Therefore, ∇V_O^1 (∇V_O^4) corresponds to the smallest (largest) oxygen gradient. We note that the 4×5 Pt/Co/O supercell is the largest system that we are able to compute with our computing power. We calculate δs_z at each layer (Fig. S13b) and show $\Sigma\delta s_z$ [= the summation of δs_z on Co layers (Co1~Co3)] as a function of the oxygen gradient in Fig. S13c. We find that $\Sigma\delta s_z$ is proportional to the lateral oxygen gradient. This demonstrates that the magnitude of δs_z increases with the lateral oxygen gradient.

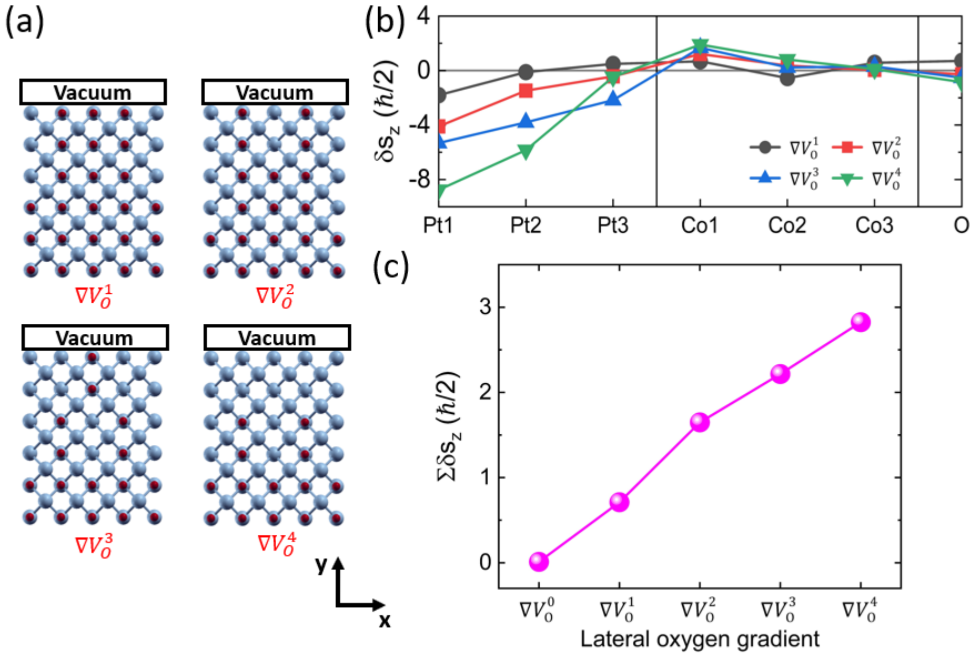

Figure S13. Z-component non-equilibrium spin density versus oxygen gradient. a, The crystal structures of the 4×5 Pt/Co/O supercell with different lateral oxygen gradients ($V_O^1 \sim V_O^4$). The red circles are the oxygen atoms and the light blue circles are the cobalt atoms. **b,** The z-component of non-equilibrium spin density δs_z with four different Pt/Co/O supercells. **c,** Summation of the δs_z over the Cobalt layer ($\Sigma\delta s_z$) with five different Pt/Co/O supercells which include the crystal structure without oxygen gradient (∇V_O^0).

Supplementary Note 14. Size dependence of z-component non-equilibrium spin density

We demonstrate below that the δs_z caused by the lateral oxygen gradient would be maintained even in a nanometer scale device. We compare two different sizes of the structures having the same lateral oxygen gradient (∇V_O^2): One is a 2×5 supercell (SC) and the other is a 4×5 supercell (see Fig. S14a). We find that δs_z values of two supercells are similar in Co layers (see Fig. S14b). This means that the size effect on δs_z is negligible once the oxygen gradient is the same. From the theoretical point of view, this is a natural result because the oxygen gradient creates a lateral electric field responsible for the Rashba effect and the magnitude of the electric field is the same for the same oxygen gradient regardless of the system size.

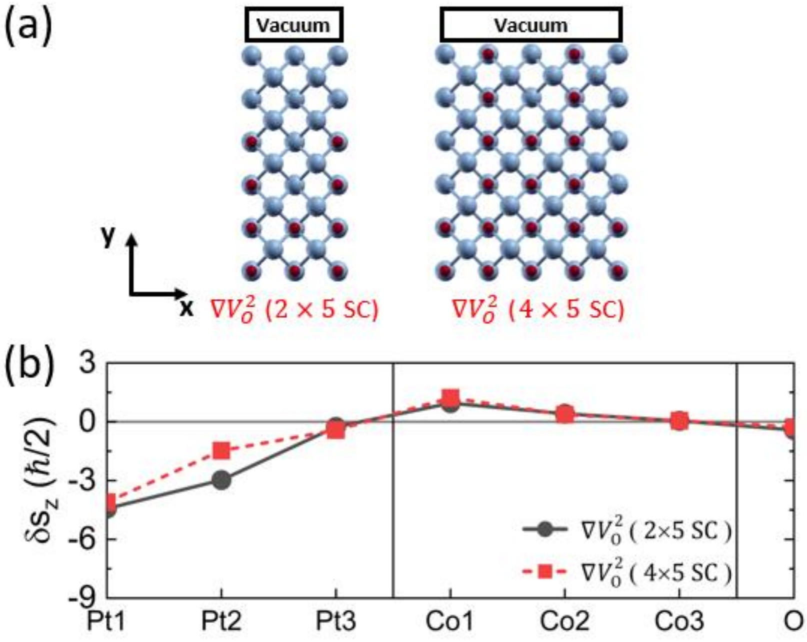

Figure S14. Size dependence of z-component non-equilibrium spin density. a, The crystal structures of two different sizes of Pt/Co/O supercell with the same lateral oxygen gradient (V_O^2). **b,** The z-component of non-equilibrium spin density (δs_z) with two different supercells.

Supplementary Note 15. Operation speed and temperature

In the original manuscript, we used a 40 nm thick gate oxide for which a gate voltage (V_G) needs to be applied for 5 minutes at 100°C to observe a noticeable electric field effect. We below show that this is not a fundamental limitation of the operation speed of our device, but it can be improved by materials engineering. To verify this argument, we performed additional experiments with a two-step approach: reducing the gate oxide thickness and introducing a double oxide gate.

First, we demonstrate that the voltage-controlled magnetic anisotropy (VCMA) effect of Pt (5 nm)/Co (1.4 nm)/AlO_x (2 nm) structures is realized even at room temperature by decreasing the thickness of the gate oxide ZrO_2 from 40 nm to 5 nm. Figure S15a shows the VCMA effect when applying a gate voltage (V_G) of 3.5 V (equivalent to 7 MV/cm) applied at *room temperature* for 5 minutes. The reduction of the coercivity (B_C) is comparable in magnitude to that in the sample with a thick ZrO_2 (40 nm) gate, where a V_G of 12 V is applied at 100°C (Fig. S15b). By simply decreasing the thickness of gate oxide, therefore, we can reduce both gate voltage (from 12 V to 3.5 V) and operation temperature (from 100°C to room temperature). We attribute this enhancement to the fact that a larger electric field is applied for a thinner gate oxide.

We further improve the VCMA effect by employing a double gate oxide of a TiO_2(2 nm)/ZrO_2(5 nm) structure, where a thin TiO_2 with high oxygen ion mobility is introduced. Figure S16a shows a similar VCMA effect (decrease in B_C) as above results by applying a *20 μs* V_G pulse (+2.5 V) at room temperature. We also find that the field-free SOT switching is achieved with this short voltage pulse of 20 μs at room temperature. Figure S16b shows the field-free switching of the Pt (5 nm)/Co (1.4 nm)/AlO_x (2 nm) sample with a TiO_2(2 nm)/ZrO_2(5 nm) gate oxide. When applying V_G to one of the gates for 20 μs at room temperature (ΔV_G =2.5 V), the sample shows a deterministic SOT switching without an in-

plane magnetic field (Fig. S16c). Furthermore, the switching polarity is reversed by changing the sign of ΔV_G, which is consistent with the results of the main text (Figures 1 and 3 in the original main text).

The above results clearly demonstrate that the operation speed of our device could be improved further through materials engineering. However, we cannot reduce the voltage pulse to less than 10 μs, which is the limit of the current experiment setup. Demonstrating faster operation (sub μs range) requires substantial work, which we think is beyond the scope of the current manuscript: a proof-of-concept demonstration of the generation of *z*-SOT by asymmetric gate voltages and associated electrical control of field-free SOT switching. Finally, we would like to note that the switching speed of an order of sub ns has been demonstrated in the ReRAM technology exploiting the local movement of the oxygen ions [S22], supporting the potential for the enhancement in the operating speed through materials development.

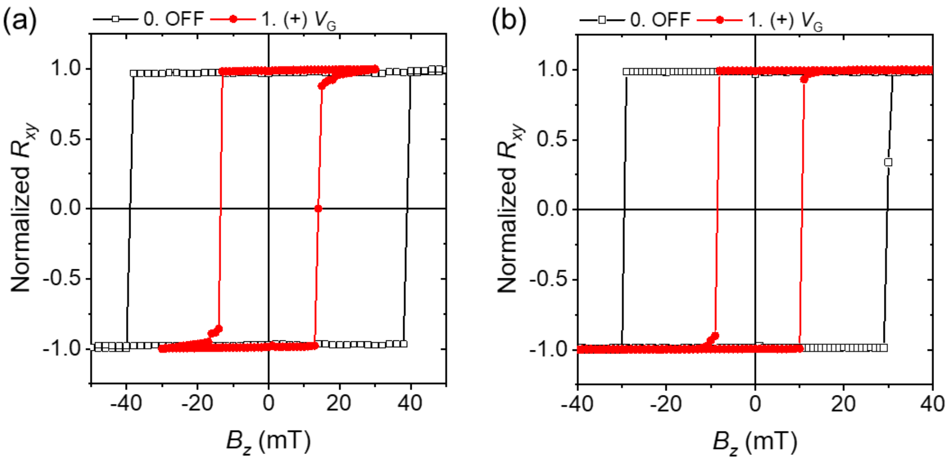

Figure S15. Voltage-controlled magnetic anisotropy with a thin gate oxide. a, Normalized anomalous Hall resistance R_{xy} as a function of perpendicular magnetic field B_z for a Pt (5 nm)/Co (1.4 nm)/AlO_x (2 nm)/ZrO_2 (5 nm) structure with a V_G of 3.5 V applied at room temperature. **b**, Normalized R_{xy} as a function of B_z for a Pt (5 nm)/Co (1.4 nm)/AlO_x (2 nm)/ZrO_2 (40 nm) sample with a V_G of 12 V applied at 100°C.

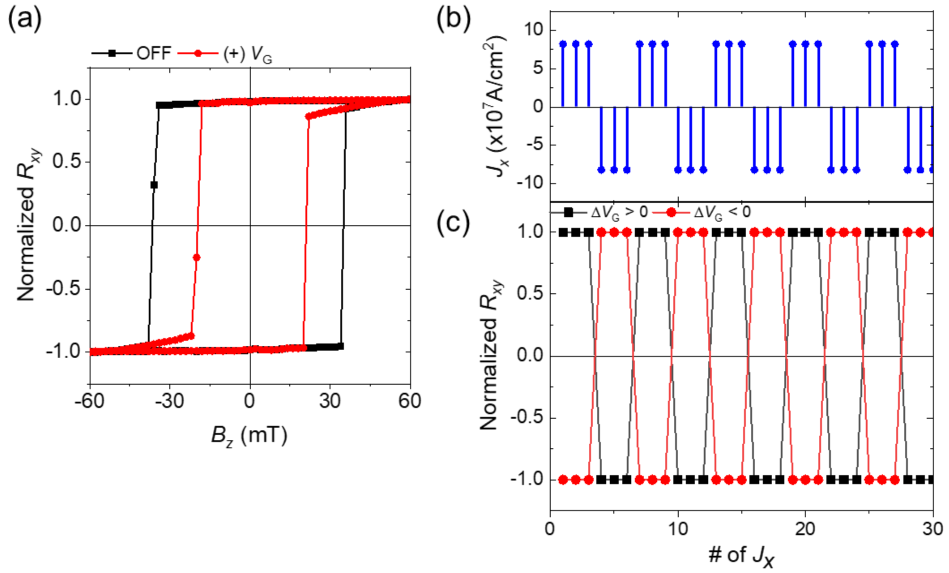

Figure S16. Voltage-controlled magnetic anisotropy with a double gate oxide. a, Normalized R_{xy} as a function of B_z in a Pt (5 nm)/Co (1.4 nm)/AlO_x (2 nm)/TiO_2(2 nm)/ZrO_2(5 nm) sample when applying a V_G pulse (+2.5 V) of 20 μs at room temperature. **b-c**, Electrical control of field-free SOT switching under a V_G pulse of 20 μs, (b) Input current pulse J_x. (c) Normalized R_{xy} vs J_x for $\Delta V_G > 0$ (black square, $V_{G,L}$=+2.5 V, $V_{G,R}$=0 V) and the $\Delta V_G < 0$ (red dot, $V_{G,L}$=0 V, $V_{G,R}$=+2.5 V).

Supplementary Note 16. Device Endurance

To test the endurance of the gate structure, we fabricate a Pt (5 nm)/Co (1.4 nm)/AlO_x (2 nm)/TiO_2 (2 nm)/ZrO_2 (5 nm)/Ru (20 nm) tunnel junction, in which a double oxide of TiO_2 (2 nm)/ZrO_2 (5 nm) is used and a Ru layer is served as a top electrode (Fig. S17a). Figure S17b shows the *I*-*V* curve of the tunnel junction, demonstrating that leakage current I_{leak} is 0.3 (-0.1) nA at a voltage pulse V_G of +2.5 (-2.5) V, at which the VCMA effect appears. To confirm the endurance of the gate operation, we repeatedly apply alternating V_G of ±2.5 V up to 1,000 times and measure the corresponding I_{leak}. Figures S17c,d show that the leakage currents remain unchanged, demonstrating the durability of the gate structure. Moreover, we measure field-dependent hysteresis loops of the anomalous Hall resistance (R_{xy}) 5 times during the repeated V_G application, as indicated by the arrows in Fig. S17d. Figures S17e,f show that the hysteresis loops are almost the same and the coercivity decreases (increases) to an almost identical value after applying a positive (negative) V_G. These results confirm that the VCMA effect is maintained and the magnetic properties are not degraded even after multiple applications of alternating V_G. We note again that the key factor to determine the endurance of the proposed SOT device is the gate-induced oxygen migration, same as the ReRAM, which demonstrates endurance over 10^{12} cycles [S23, S24].

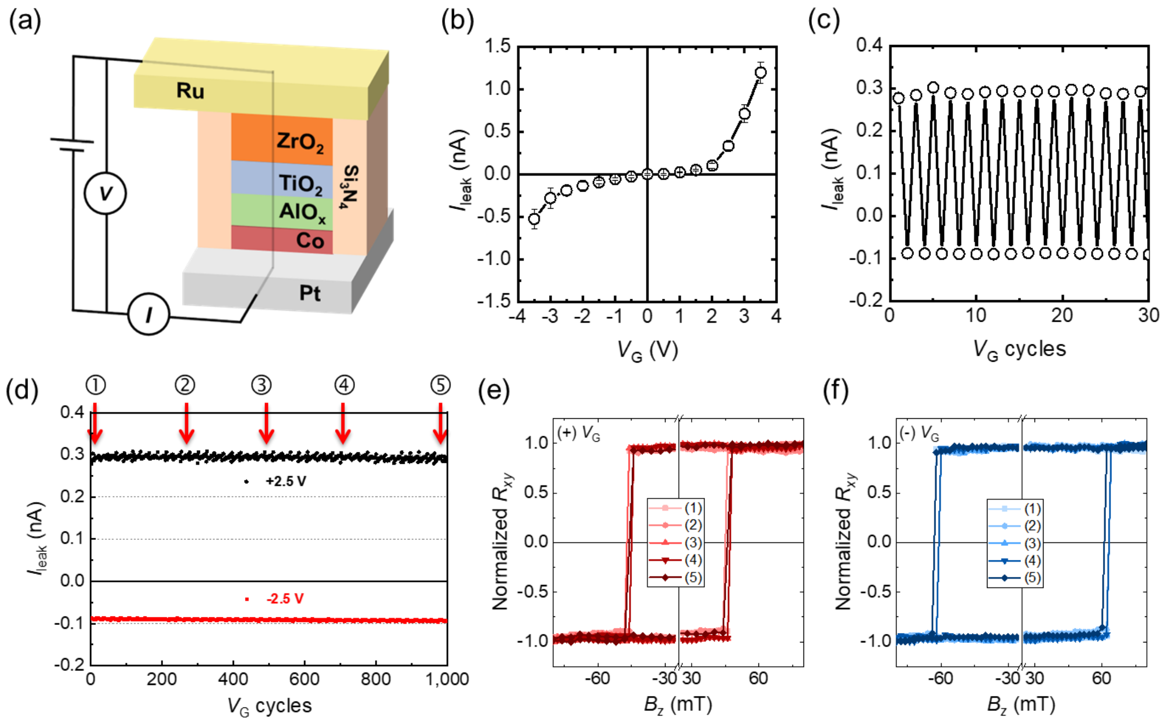

Figure S17. Endurance of VCMA effect. a. Schematic of a device structure for endurance test. **b,** *I-V* characteristics in a Pt/Co/AlO_x/TiO_2/ZrO_2 tunnel junction. The error bars correspond to the standard deviation of the mean value of I_{leak} measured 10 times. **c-d,** I_{leak} versus a number of gate voltage V_G voltage of ±2.5 V with a 1 ms pulse width for first 30 cycles (c) and 1000 cycles (d). **e-f**, Normalized R_{xy} as a function of B_z in the Pt/Co/AlO_x/TiO_2/ZrO_2 sample measured after applying a V_G of +2.5 V (e) and -2.5 V (e), as indicated by the arrows in Fig. S17d

Supplementary Note 17. SPICE circuit simulation

We additionally perform the feasibility analysis of our device using SPICE circuit simulations that demonstrates the operation of an XOR gate based on the lateral SOT device, which is compared with existing CMOS logic gates. Figure S18a shows the schematic of a device design consisting of a magnetic tunnel junction (MTJ) on the left and a single side gate on the right, where the width of the MTJ electrode is 40 nm and that of the gate electrode is 30 nm. We note that this design has no practical difficulty to be realized with conventional semiconductor processes. Several distinct features of this device design are as follows.

First, we note that two side gates are not necessary but a single gate is sufficient for achieving the field-free SOT switching, as demonstrated in Fig. S18 where the gate voltage is applied to only a single side gate. Therefore, the application of V_G on a single side gate controls SOT switching of the free layer, of which direction is read via the tunnel magnetoresistance (TMR) of the MTJ. Second, in this device, the gating part on the right is separated from the reading (TMR) part on the left so that a possible voltage-induced modulation of electrical properties of the gate does not affect the reading signal. Then a remaining question is if this lateral SOT device is competitive with existing silicon technologies in terms of the size (area) and the energy consumption. Utilizing the state-of-art circuit simulation, we show below that our SOT device is indeed competitive with existing silicon technologies.

To show the feasibility of our device, we design an XOR gate consisting of 3 transistors and 1 lateral SOT device (1 MTJ with a side gate) using 28 nm CMOS technology. Here, the output of the logic gate, the resistance of the MTJ (R_{MTJ}), is determined by two parameters of the gate voltage (V_G) and input current (I_{IN}). Accordingly, we performed the layout design to estimate the area and the HSPICE circuit simulation to estimate the energy consumption of the proposed logic gate. Figure S18b illustrates the schematic and layout design of the lateral SOT-based XOR gate. Here, the lateral SOT device is placed between the metal 2 (M2) and the metal

3(M3) facing downward; the heavy metal (HM) is placed on the top of the MTJ; and a side gate is placed on the bottom of the MTJ. Furthermore, both the top electrode of the MTJ and the gate electrode are in contact with the metal 2 (M2) lines connected to the transistors. As a result, the MTJ (or lateral SOT device) is located on top of the transistor as in commercialized MRAMs. Notably, the size of the lateral SOT device is much smaller than that of the transistor; the size of MTJ is only about 5% of that of the total XOR gate. So, the total device area is predominantly determined by the number of transistors used in the logic gate. The proposed XOR gate consisting of 3 transistors has much smaller device area than the CMOS XOR gate that requires 12 transistors (Fig. S18c); according to the layout design, the proposed XOR gate shows an area of 0.1995 μm^2, ~23% of that of the CMOS XOR gate (Fig. S18d). Therefore, the lateral SOT device is beneficial in terms of the device area, which is one of the most important considerations for semiconductor industries to choose a new emerging device because the device area affects the production cost in a direct way.

We also carry out the HSPICE circuit simulation to estimate the energy consumption of the proposed logic gate. The waveform shown in Fig. S19a demonstrates the operation of the lateral SOT XOR gate. Here, V_G and I_{IN} are two inputs; positive input values (+0.5 V and +173 μA) represent digital input '1' and negative input values (-0.5 V and -173 μA) represent '0'. Note that the MTJ is initialized as the parallel alignment of magnetization or low resistance state (digital output '0'). When two inputs are of different signs, the free layer of the MTJ is switched, resulting in a high resistance state, and digital output '1'. Otherwise, the R_{MTJ} remains as the low resistance state or digital output '0'. We also simulated the energy consumption of the lateral SOT XOR gate as a function of input current density and compared with those of the CMOS XOR gates with an equivalent CMOS technology node. Fig. S19b shows that the proposed lateral SOT XOR gate can have a comparable energy consumption to the CMOS XOR gates, when the SOT switching can be operated at a current density of less than

1×10^7 A·cm^{-2} and a current duration of sub ns [S25]. Here, we note that the energy consumption for gate operation (a leakage current of sub nA) is negligible. The material parameters for the simulations are shown in Table S2.

We finally note that our spin logic can be applied to Field Programmable Gate Arrays (FPGA). Since the current state-of-the-art CMOS-based FPGA also takes about 5-10 minutes for reconfiguration, long gating time of tens of μs is not an issue in this case.

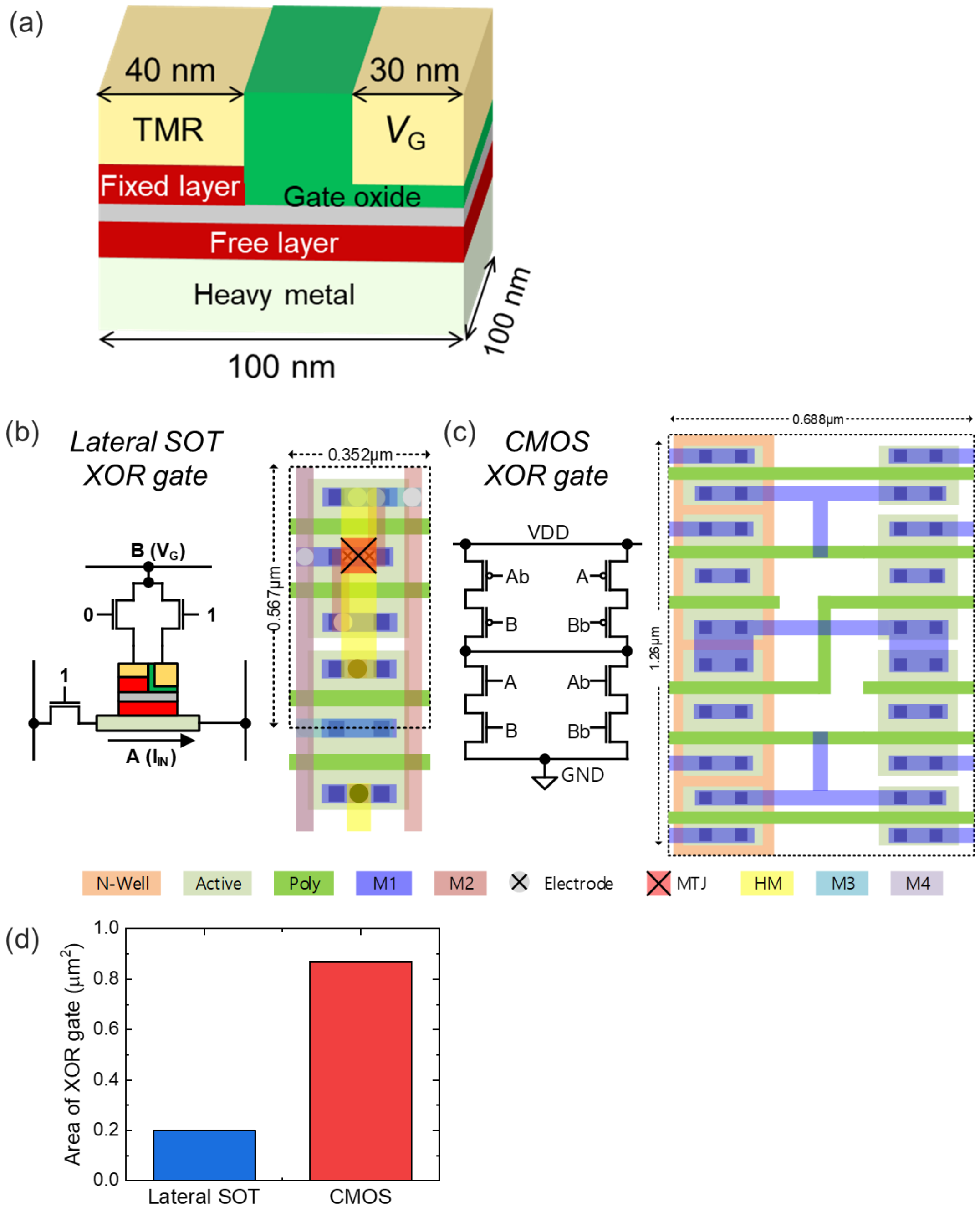

Figure S18. Simulation of XOR logic gate using a lateral SOT device for size comparison with CMOS. a, Schematic of MTJ with a single side gate. **b,** Schematics and layout of a lateral SOT XOR gate. **c,** Schematics and layout of a CMOS XOR gate. **d,** Area comparison of XOR gate using the lateral SOT device or conventional CMOS.

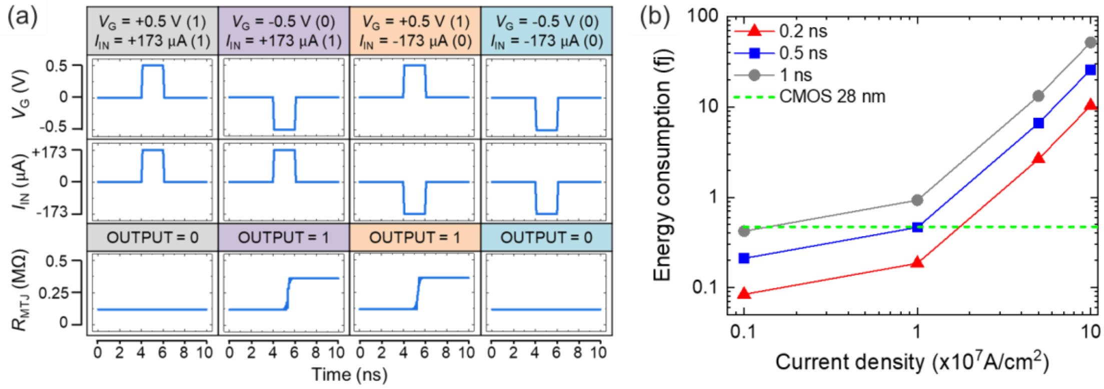

Figure S19. Simulation of XOR logic gate using a lateral SOT device for energy consumption comparison with CMOS. a, Waveform of XOR gate operation using a lateral SOT with a side gate. **b,** Energy consumption (per switching) of the lateral SOT XOR gate as a function of current density with different current durations, compared with those of the CMOS XOR gate with the CMOS 28 nm technology node.

Table R2. Material Parameters for the SPICE simulations.

Parameter	Value
Ms of free layer	1000 emu/cm^3
Thickness of free layer	1 nm
Damping parameter of free layer	0.01
PMA constant	8.3×10^6 erg/cm^3
HM resistivity	1.2×10^{-7} Ωcm
Thickness of HM layer	5 nm
MTJ size	100 nm × 100 nm
RA of MTJ	480 Ωμm^2
TMR ratio	150 %

References

[S1] Tan, H., Verbeeck, J., Abakumov, A. & Van Tendeloo, G. Oxidation state and chemical shift investigation in transition metal oxides by EELS. *Ultramicroscopy* **116**, 24–33 (2012).

[S2] Garello, K. *et al.* Symmetry and magnitude of spin-orbit torques in ferromagnetic heterostructures. *Nat. Nanotechnol.* **8**, 587–593 (2013).

[S3] Yu, G. *et al.* Switching of perpendicular magnetization by spin-orbit torques in the absence of external magnetic fields. *Nat. Nanotechnol.* **9**, 548 (2014).

[S4] Lee, D.-K. & Lee, K.-J. Spin-orbit torque switching of perpendicular magnetization in ferromagnetic trilayers. *Sci. Rep.* **10**, 1772 (2020)

[S5] Hayashi, M., Kim, J., Yamanouchi, M. & Ohno, H. Quantitative characterization of the spin-orbit torque using harmonic Hall voltage measurements. *Phys. Rev. B* **89**, 144425 (2014).

[S6] Baek, S. C. *et al.*Spin currents and spin-orbit torques in ferromagnetic trilayers. *Nat. Mater.* **17**, 509–513 (2018).

[S7] C.J. Durrant. *et al.* Scanning Kerr microscopy study of current-induced switching in Ta/CoFeB/MgO films with perpendicular magnetic anisotropy. *Phys. Rev. B* **93**, 014414 (2016).

[S8] Avci, C. O. *et al.* Interplay of spin-orbit torque and thermoelectric effects in ferromagnet/normal-metal bilayers. *Phys. Rev. B* **90**, 224427 (2014).

[S9] Maruyama, T. *et al.* Large voltage-induced magnetic anisotropy change in a few atomic layers of iron. *Nat. Nanotechnol.* **4**, 158–161 (2009).

[S10] Bauer, U. *et al.* Magneto-ionic control of interfacial magnetism. *Nat. Mater.* **14**, 174–181 (2015).

[S11] Park, K. W. *et al.* Electric field control of magnetic anisotropy in the easy cone state of Ta/Pt/CoFeB/MgO structures. *Appl. Phys. Lett.* **109**, 012405 (2016).

[S12] Ozaki, T. Variationally optimized atomic orbitals for large-scale electronic structures. *Phys. Rev. B* **67**, 155108 (2003).

[S13] Bachelet, G. B. *et al.* Pseudopotentials that work: From H to Pu. *Phys. Rev. B* **26**, 4199 (1984).

[S14] Perdew, John P. *et al.* Generalized gradient approximation made simple. *Phys. Rev. Lett.* **78**, 1396 (1997).

[S15] Negele, J. W. & Orland, H. Quantum many-particle systems. Perseus Books Publishing, L.L.C. (1998).

[S16] Datta, S. Nanoscale device modeling: the Green's function method. *Superlattices and Microstrut.* **28**, 4 (2000).

[S17] Datta, S. Electronic transport in mesoscopic systems (Cambridge University Press, Cambridge, 1997).

[S18] Bychkov, Y. A. & Rashba, E. I. Oscillatory effects and the magnetic susceptibility of carriers in inversion layer. *J. Phys. C* **17**, 6039 (1984).

[S19] Krupin, O. *et al.* Rashba effect at magnetic metal surfaces. *Phys. Rev. B* **71**, 201403(R) (2005).

[S20] Park, J.-H. *et al.* Orbital chirality and Rashba interaction in magnetic band. *Phys. Rev.* ***B*** **87**, 041301(R) (2013).

[S21] Grytsyuk, S. *et al.* k-asymmetric spin splitting at the interface between transition metal ferromagnets and heavy metals. *Phys. Rev. B* **93**, 174421 (2016).

[S22] Wang, C. *et al.* Ultrafast RESET Analysis of HfOx-Based RRAM by Sub-Nanosecond Pulses. *Adv. Electron. Mater.* **3**, 1–6 (2017).

[S23] Hsu, C., Wang, I., Lo, C., Chiang, M. & Jang, W. Self-Rectifying Bipolar TaO x / TiO 2 RRAM with Superior Endurance T167. *2013 Symp. VLSI Circuits 166–167 (2013).*

[S24] Prakash, A. *et al.* Demonstration of low power 3-bit multilevel cell characteristics in a TaOx-Based RRAM by stack engineering. *IEEE Electron Device Lett.* ***36**, 32–34 (2015).*

[S25] Cubukcu, M. *et al.* Ultra-Fast Perpendicular Spin – Orbit Torque MRAM, *IEEE Trans. Magn.* **54**, 4–7 (2018).
